# The efficacy of human epidermal growth factor receptor 2 (HER2) blockade switching mode in refractory patients with HER2-positive metastatic breast cancer: a phase II, multicenter, single-arm study (SYSUCC-005)

**DOI:** 10.1186/s12885-022-09399-2

**Published:** 2022-03-15

**Authors:** Fangfang Duan, Muyi Zhong, Yuyu Ma, Chenge Song, Lehong Zhang, Ying Lin, Zhiyong Wu, Yuanqi Zhang, Jiajia Huang, Fei Xu, Yanxia Shi, Shusen Wang, Zhongyu Yuan, Wen Xia, Xiwen Bi

**Affiliations:** 1 Department of Medical Oncology, Sun Yat-Sen University Cancer Center, The State Key Laboratory of Oncology in South China, Collaborative Innovation Center for Cancer Medicine, 651 Dongfeng Road East, Guangdong 510060 Guangzhou, China; 2 Department of Breast Oncology, Dongguan People’s Hospital, Dongguan, Guangdong China; 3 Department of Breast Oncology, The Second Affiliated Hospital of Guangzhou Medical University, Guangzhou, Guangdong China; 4 Department of Breast Oncology, The First Affiliated Hospital of Sun Yat-Sen University, Guangzhou, Guangdong China; 5 Department of Breast Oncology, Shantou Central Hospital, Shantou, Guangdong China; 6 Department of Vascular Thyroid Breast Surgery, The Affiliated Hospital of Guangdong Medical University, Zhanjiang, Guangdong China

**Keywords:** HER2-positive breast cancer, Trastuzumab refractory, Lapatinib, HER2 blockade therapy

## Abstract

**Background:**

Despite significant survival improvement in human epidermal growth factor receptor 2 (HER2) blockade for HER2-positive breast cancer, resistance to anti-HER2 remains inevitable. Subsequent anti-HER2 with continuing trastuzumab beyond progression is acceptable with limited efficacy when other anti-HER2 treatment is unavailable. This single-arm, phase II study (SYSUCC-005) aimed to explore the efficacy of switching mode for HER2-positive refractory metastatic breast cancer.

**Methods:**

Patients with HER2-positive metastatic breast cancer rapidly progressing during pre-trastuzumab from six hospitals in China were designed to switch to lapatinib 1,250 mg orally once per day continuously plus capecitabine (1,000 mg/m^2^ orally twice per day on days 1–14) or vinorelbine (25 mg/m^2^ intravenously once per day on days 1 and 8) of each 21-day cycle. The primary endpoint was progression-free survival (PFS).

**Results:**

Between January 5, 2015 and May 31, 2020, 159 patients were eligible in this study. The median follow-up was 33.1 months, a median PFS of 8.5 months was achieved. Brain metastases (hazard ratio [HR] = 1.582, 95% confidence interval [CI] 1.019- 2.453, *P* = 0.041) and ≥ 2 metastatic sites (HR = 1.679, 95% CI 1.151–2.450, *P* = 0.007) were independent prognostic factors for PFS. The most common grade ≥ 3 adverse events were diarrhea (3.8%) and hand-foot syndrome (9.4%).

**Conclusion:**

The switching mode showed predominant efficacy, which might be a prior therapeutic option over continuing mode in subsequent anti-HER2 therapy for patients with HER2-positive refractory metastatic breast cancer.

**Trial registration:**

This trial was registered on ClinicalTrials.gov (NCT02362958) on 13/02/2015.

## Introduction

Amplification of human epidermal growth factor receptor 2 (HER2) occurs in nearly 25% of all breast cancer types and enhances its aggressiveness [1, 2]. Trastuzumab, a humanized murine IgG monoclonal HER2 antibody, has significantly improved the survival of patients with HER2-positive metastatic breast cancer [3, 4]. In the first- line setting, trastuzumab and pertuzumab plus taxane is recommended as the standard care for HER2-positive metastatic breast cancer [5, 6]. However, de novo or acquired resistance to anti-HER2 in inevitable, although subsequent anti-HER2 with continuing trastuzumab beyond progression is acceptable when other anti-HER2 treatment unavailable, its survival efficacy is limited [7]. Trastuzumab emtansine (TDM1) is generally recommended as the standard second-line care by National Comprehensive Clinical Network (NCCN) guidelines for patients rapidly progressing during previous trastuzumab-based treatment [8, 9]. But in developing area including China, TDM1 in second-line metastatic setting is difficult to afford due to limited medical insurance, continuing trastuzumab plus chemotherapy remains to be recommend as an appropriate intervention.

Lapatinib (Tykerb), an orally active, small molecule, reversible tyrosine kinase inhibitor targeting HER2, has been proven to not have cross-resistance with trastuzumab [10, 11]. For patients rapidly developing progression during previous trastuzumab-based regimens, continuing anti-HER2 with trastuzumab or switching to lapatinib plus chemotherapeutic drugs was proven effective and recommended as available interventions in second-line setting instead of TDM1 [12–15]. However, no compelling and sufficient evidence indicates which one is more effective. Given these findings, we conducted this study (SYSUCC-005) to explore the efficacy and safety of switching treatment mode in patients with HER2-positive refractory metastatic breast cancer.

## Methods

### Study design

The SYSUCC-005 trial (NCT02362958) is an open-label, multicenter, phase II, single-arm study for evaluating the efficacy and safety of switching anti-HER2 therapy mode in women with HER2-positive refractory metastatic breast cancer who rapidly progressed during prior adjuvant/first-line trastuzumab, which was conducted in accordance with the Declaration of Helsinki and good clinical practice (GCP) guidelines. The ethics committee of Sun Yat-sen University Cancer Center (SYSUCC) reviewed and approved this study protocol before implementation, and every patient provided written informed consent prior to enrollment.

### Patient eligibility

Eligible patients were females aged 18 to 75 years with pathologically confirmed metastatic breast cancer. HER2 positivity refers to a score of 3 + or 2 + by immunohistochemistry (IHC) with ERBB2 gene amplification on fluorescence in situ hybridization (FISH). Patients were required to have 0 or 1 Eastern Cooperative Oncology Group (ECOG) performance status, and measurable disease according to Response Evaluation Criteria in Solid Tumors (RECIST) version 1.1 [16]. Patients were considered refractory to trastuzumab if they recurred during or within 12 months after completing adjuvant trastuzumab or rapidly progressed in the first radiological evaluation during first-line trastuzumab for metastatic disease [17]. A life expectancy of at least 12 weeks, adequate hematologic, hepatic, renal, and cardiac function, and prior chemotherapy with anthracycline and/or taxane were required. Key exclusion criteria included left ventricular ejection fraction (LVEF) < 45% by echocardiogram; uncontrolled medical problems, i.e., arrhythmia, myocardial infarction, dysphrenia; and ineligibility for this study assessed by investigators.

### Treatment and evaluations

Patients were designed to receive lapatinib 1,250 mg orally once per day continuously plus capecitabine (1,000 mg/m^2^ orally twice per day on days 1–14) or vinorelbine (25 mg/m^2^ intravenously once per day on days 1 and 8) for each 21-day cycle. Dose modifications and delays due to treatment-related toxicity were allowed. Lapatinib was suspended for up to two weeks for any grade ≥ 3 toxicity, an initial dose of 1250 mg/d could be resumed after recovery from grade ≤ 3 adverse events (AEs), but dose reductions from 1,250 mg to 1,000 mg to 750 mg was required in cases of grade 4 AEs and lapatinib would be discontinued permanently if interstitial pneumonitis or cardiac dysfunction of grade ≥ 3 occurred. The dose of capecitabine was permitted to be reduced stepwise by 25% after grade ≥ 2 hand-foot syndrome (HFS), even though patients fully recovered from it, and it would be discontinued permanently if grade ≥ 2 HFS occurred three times or more.

Tumors were evaluated at baseline, every three cycles during the intervention by computed tomography (CT) or magnetic resonance imaging (MRI) according to the RECIST (version 1.1) until radiographically confirmed disease progression, initiation of new anticancer therapy, or subject discontinuation of the study (e.g., death, subject’s request, loss to follow-up). AEs were assessed and graded according to the National Cancer Institute’s Common Toxicity Criteria for Adverse Events (version 4.0).

### Endpoints

The primary endpoint was progression-free survival (PFS), defined as the time from initiation of intervention in this study to disease progression or death due to any cause. Secondary endpoints included overall survival (OS), defined as the time from initiation of treatment to death due to any cause; response rate (RR), defined as the proportion of patients with a complete response (CR) or partial response (PR); clinical benefit rate (CBR), defined as the proportion of patients with CR, PR, and stable disease (SD) for ≥ 24 weeks; and safety.

### Statistical analysis

The median PFS reached 5.6 months for patients with HER2-positive refractory metastatic breast cancer continuing trastuzumab beyond prior progression based on the prospective phase III study LUX-Breast 1 [18]. We assumed that patients in this SYSUCC-005 study achieved a median PFS of 8.1 months, i.e., an absolute increase of 2.5 months, given an 80% statistical power at a two-sided significance level of 0.05, at least 159 patients were required including a 10% dropout rate.

Categorical variables were shown as frequencies with percentages, and continuous data were provided as mean with range. We used the Kaplan–Meier method to estimate cumulative survival probabilities. Univariate and multivariate Cox proportional hazards regression models, which have been verified according to the Schoenfeld residuals [19], were performed to determine the hazard ratios (HRs) with 95% confidence interval (CI) and identify prognostic factors. Only achieving a *P* value of < 0.2, variables were further incorporated into the multivariate model. All statistical tests were two-sided, and *P* < 0.05 was considered statistically significant. All analyses were performed with SPSS (version 22.0) and R (version 4.0.1).

## Results

### Patient characteristics

**Fig. 1:**
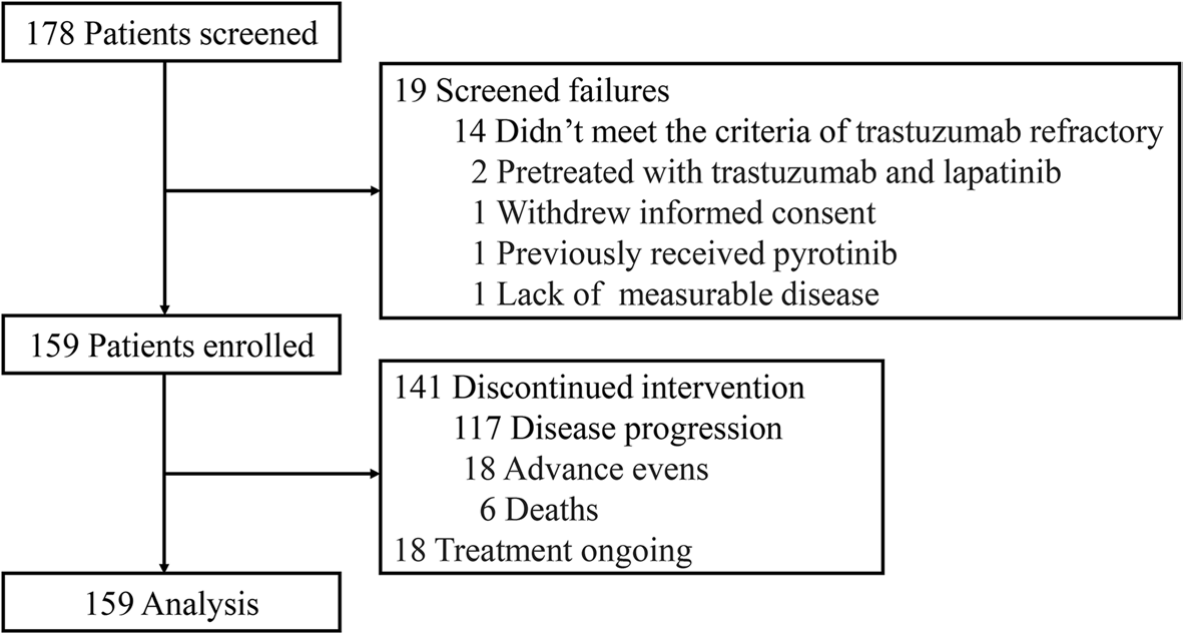
Flow chart of patients through this SYSUCC-005 trial

178 women with HER2-positive metastatic breast cancer from six hospitals in China were screened between January 5, 2015 and May 31, 2020, of whom, 19 patients were ineligible due to various causes as shown in Fig. 1, the most common reason for screen failure was not conforming to the definition of trastuzumab refractory (14 patients). Finally, 159 patients were enrolled in this study, 141 of them discontinued intervention due to disease progression, unacceptable toxicities, or death.

As listed in Table 1, the mean age was 47.3 years old (range 24 to 75). 47.2% of patients (75/159) were premenopausal and 66.0% of patients (105/159) were considered as hormone receptor (HR) negative. More than half of patients (63.5%) had visceral metastases, and 23.3% of patients had brain metastases. 32.7% of subjects (52/159) previously received trastuzumab only in the adjuvant setting, and 51.6% of cases (82/159) received trastuzumab as the first-line care of metastatic disease plus chemotherapeutic drugs, including 56.1% (46/82) taxane, 13.4% (11/82) vinorelbine, 11.0% (9/82) capecitabine, 7.3% (9/82) gemcitabine, 12.2% (10/82) others, respectively. A duration of previous trastuzumab less than 6.0 months was reported for 44.7% of patients (71/159). Capecitabine and vinorelbine were delivered in 67.9% (108/159) and 32.1% (51/159) of patients, respectively.

**Table 1:** Patient characteristics at baseline

Characteristics	Mean or Case Number (%)
**Age (years) at diagnosis**	
Mean	47.3
Range	24–75
**Menopausal status at diagnosis**	
Premenopausal	75 (47.2)
Postmenopausal	84 (52.8)
**HR status**	
Negative	105 (66.0)
Positive	54 (34.0)
**Number of metastatic sites**	
< 2	91 (57.2)
≥ 2	68 (42.8)
**Brain metastases**	
Yes	37 (23.3)
No	122 (76.7)
**Visceral metastases**^**a**^	
Yes	101 (63.5)
No	58 (36.5)
**Prior trastuzumab treatment**	
Adjuvant setting only	52 (32.7)
Adjuvant and metastatic setting	25 (15.7)
Metastatic setting only	82 (51.6)
**Duration of previous trastuzumab**	
< 6 months	71 (44.7)
≥ 6 months	88 (55.3)
**Chemotherapy regimens with Lapatinib**	
Capecitabine	108 (67.9)
Vinorelbine	51 (32.1)

^a^Metastases in lung/liver

*Abbreviation*: *HR* hormone Receptor

### Efficacy

**Fig. 2:**
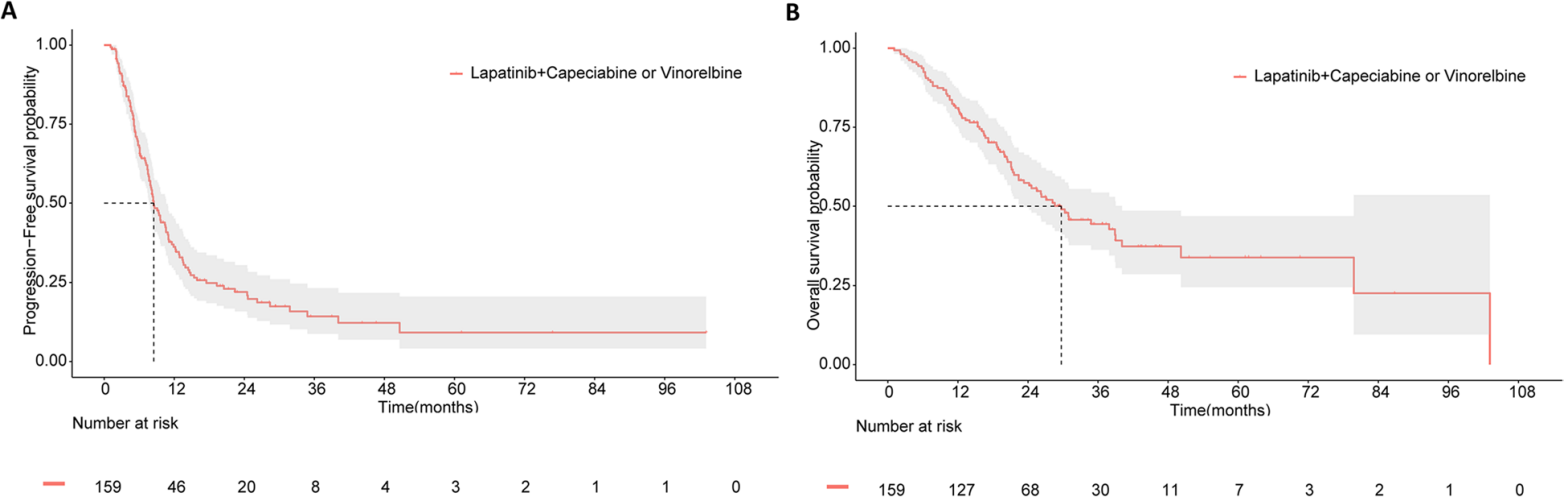
Kaplan–Meier curves for all patients enrolled in this trial. **A:** The Kaplan–Meier curve for progression-free survival (PFS). **B:** The Kaplan–Meier curve for overall survival (OS)

The cutoff date was November 03, 2020, after a median follow-up of 33.1 months, 117 disease progression events and 82 death events were observed. The median PFS and OS were 8.5 months (95% CI 7.2–9.8) and 29.7 months (95% CI 21.9–37.5), respectively (Fig. 2A and B). Additionally, exploratory analysis of PFS and OS by the multivariate Cox regression method (Table 2) indicated that brain metastases (PFS: HRs = 1.582, 95% CI 1.019–2.453, *P* = 0.041; OS: HRs = 1.833, 95% CI 1.133–2.630, *P* = 0.013) and ≥ 2 metastatic sites (PFS: HRs = 1.679, 95% CI 1.151- 2.450, *P* = 0.007; OS: HRs = 1.691, 95% CI 1.087–2.630, *P* = 0.020) remained to be independent prognostic factors.

**Table 2:** Multivariate Cox regression analysis of PFS and OS

Characteristics	PFS		OS	
	**Hazard ratios (95%CI)**	***P* value**	**Hazard ratios (95%CI)**	***P* value**
**Age (year)**				
≤ 45	1.0			
> 45	1.558 (1.054–2.302)	0.026*		
**Menopausal status**				
Premenopausal	1.0			
Postmenopausal	0.745 (0.438–1.270)	0.280		
**Number of metastatic sites**				
< 2	1.0		1.0	
≥ 2	1.679 (1.151–2.450)	0.007*	1.691 (1.087–2.630)	0.020*
**Brain metastases**				
No	1.0		1.0	
Yes	1.582 (1.019–2.453)	0.041*	1.833 (1.133–2.963)	0.013*
**Duration of prior trastuzumab treatment**				
< 6 months			1.0	
≥ 6 months			0.863 (0.546–1.364)	0.528

^*^P < 0.05

^a^Metastases in lung/liver

*Abbreviation*: *PFS* Progression-Free Survival, *OS* Overall Survival, *CI* Confidence Interval

Among all evaluable 159 patients, the RR was 29.6% (95% CI 22.6–37.3). No patients obtained a CR. 47 (29.6%), 51 (32.1%), and 24 (15.1%) patients showed a PR, SD for ≥ 24 weeks, and PD, respectively. The CBR among all patients in current study was 61.7%.

## Safety

Treatment-related AEs are shown in Table 3. The most frequent AEs were diarrhea and HFS, but grade 3 or higher AEs were just observed in 3.8% and 9.4% of patients, respectively. 11.3% (18/159) discontinued lapatinib due to AEs. No treatment-related deaths or life-threatening AEs were observed.

**Table 3:** Adverse Events

Adverse events in all cycles	Number of patients (%)	
	All grade	Grade ≥ 3
Diarrhea	57 (35.8)	6 (3.8)
Stomatitis	7 (4.4)	1 (0.6)
Hand-foot syndrome	28 (21.7)	15 (9.4)
Vomiting	7 (4.4)	0
Rash	23 (14.5)	0
Myelosuppression	15 (9.4)	9 (5.7)
Nausea	1 (0.6)	0
Peripheral neurotoxicity	10 (6.3)	0
Malaise	1 (0.6)	0

## Discussion

In current study, we demonstrated that switching mode with lapatinib-based treatment as subsequent anti-HER2 care for HER2-positive trastuzumab refractory metastatic breast cancer achieved a clinical efficacy.

The phase III trial GBG26 and the observational Hermine study both found that although continuing trastuzumab beyond previous progression could significantly improve the median time to progression (TTP), no significant improvement in OS was observed [12, 13]. Whereas, other two multicenter phase III trials EGF100151 [14] and ALTERNATIVE [20] showed that switching to lapatinib yielded a median TTP/PFS superior to that of chemotherapy alone or continuing trastuzumab in refractory patients with HER2-positive metastatic breast cancer. Our study found that female patients with HER2-positive trastuzumab refractory metastatic breast cancer switching to lapatinib achieved a median PFS of 8.5 months, which was consistent with these two studies [14, 20], and reached to the original assumes at design compared with those continuing trastuzumab in the LUX-Breast 1 study, which was also especially designed for HER2-positive trastuzumab refractory metastatic breast cancer. Moreover, a superior median OS in this SYSUCC-005 study was also achieved [18]. These suggested that subsequent anti-HER2 with switching mode might be preferred over continuing mode with trastuzumab for HER2-positive trastuzumab refractory metastatic breast cancer.

A phase II clinical study, which compared trastuzumab plus capecitabine with lapatinib plus capecitabine in patients with HER2-positive metastatic breast cancer previously treated with trastuzumab, failed to find significant differences in PFS and OS between these two groups. The small sample size and the fact that two-thirds of patients were nonrefractory (prior to trastuzumab naive or duration of prior systemic treatment ≥ 1 year) might explain the inconsistent findings [21]. Interestingly, although a 61.7% CBR of switching to lapatinib in this study was achieved, the RR was just 29.6%. It suggested that lapatinib might be more effective in disease control rather than tumor shrinkage, which was in line with the therapeutic goal of salvage management of metastatic breast cancer [12].

Besides, we performed exploratory analysis to exploit potentially independent factors of HER2-positive trastuzumab refractory metastatic breast cancer patients switching to lapatinib. The multivariate Cox analysis in current SYSUCC-005 study demonstrated that brain metastases was a significant indicator for both PFS and OS. Two previous prospective phase II studies have demonstrated that patients with brain metastases from HER2-positive refractory metastatic breast cancer could significantly benefit from switching to lapatinib plus chemotherapy [22, 23]. As is known to us that brain metastasis occurs in 30%-50% of HER2-positive metastatic breast cancer and trastuzumab is unable to cross the blood–brain barrier [24, 25], nearly one-third of patients with HER2-positive breast cancer ultimately develop brain metastases after previous trastuzumab, and different mechanisms of action between trastuzumab and lapatinib [10, 12], switching mode with lapatinib might be a sensible therapeutic option for those with brain metastases. In addition, patients with ≥ 2 metastatic sites, indicating a higher tumor burden, are possible candidates for switching to lapatinib, which has been proven in previous studies [22, 26].

For patients with HER2-positive metastatic breast cancer rapidly progressing during previous trastuzumab-based treatment, NCCN guidelines have modified early therapeutic lines of metastatic breast cancer and recommend several therapeutic options for subsequent anti-HER2 treatment owing to the conception of therapeutic hierarchy and the development of other more efficient options, including continuing trastuzumab with other chemotherapeutic drugs, dual-targeted therapy containing trastuzumab plus pertuzumab or lapatinib, administering TDM1, and switching to lapatinib plus chemotherapy [8]. Unfortunately, patients in developing areas including China have limited funding for medical care, and TDM1 is unavailable in second or later line setting in those regions. Therefore, administration of dual-targeted therapy or TDM1 for them is unrealistic in clinical practice, thereby lapatinib or trastuzumab plus a chemotherapy regimen remains the preferred therapeutic options. Our study accurately suggested that switching to lapatinib plus chemotherapy could achieve a satisfactory PFS and OS for patients with HER2-positive refractory metastatic breast cancer, which was somehow confirmed in the CEREBEL (EGF111438) study that combing lapatinib with capecitabine after disease progression on previous trastuzumab-based regimen was advisable for HER2-positive metastatic breast cancer [27]. Moreover, we identified independent characteristics as potential predictors for survival derived from this switching mode therapy, which might improve the efficacy of salvage treatments for HER2-positive trastuzumab refractory metastatic breast cancer patients and provide potential guidance for designing clinical studies exploring latter lines of therapy in future.

Several limitations in this study should be noted. First, it is a single-arm phase II study limited to several medical centers in China, so extrapolation of our outcomes to clinical practice should be done with caution. Second, this trial was limited to recruitment at Chinese hospitals, results may not be generalizable to patients from other geographic regions and with other racial/ethnic backgrounds. Some studies have been carried out to evaluate differences in treatment responses and survival outcomes among patients of different racial groups [28, 29]. Third, we started this study before pertuzumab clinically available in China, so we failed to explore the efficacy of this switching mode beyond failure during previous dual anti-HER2 therapy with trastuzumab and pertuzumab. Although we adopted a regular drug in this trial, the switching mode for therapeutic conception remained commendable at the time of designing, when continuing trastuzumab beyond progressing during previous trastuzumab was recommended for HER2-positive refractory metastatic breast cancer. Thus, the quality of current study was its switching mode for therapy, which was also clinically appropriated for salvaging HER2-positive trastuzumab refractory breast cancer nowadays.

## Conclusion

In conclusion, HER2 blockade switching mode with lapatinib might be a prior therapeutic option over continuing mode with trastuzumab beyond progression during prior trastuzumab-based regimens, as the switching strategy achieving satisfactory efficacy in patients with HER2-positive, trastuzumab refractory metastatic breast cancer.

## Data Availability

The authenticity of this article has been validated by uploading the key raw data onto the Research Data Deposit public platform (www.researchdata.org.cn), with the approval RDD number, RDDA2021001961.

## Abbreviations

HER2: Human epidermal growth factor receptor type 2
HR: Hormone receptor
TDM1: Trastuzumab emtansine
NCCN: National Comprehensive Clinical Network
GCP: Good clinical practice
IHC: Immunohistochemical
FISH: Fluorescence in situ hybridization
ECOG: Eastern Cooperative Oncology Group
RECIST: Response Evaluation Criteria in Solid Tumors
LVEF: Left ventricular ejection fraction
AEs: Adverse events
HFS: Hand foot syndrome
PFS: Progression-free survival
OS: Overall survival
RR: Response rate
CR: Complete response
PR: Partial response
SD: Stable disease
CBR: Clinical benefit rate
CI: Confidence interval
HRs: Hazard ratios
TTP: Time to progression
ASCO: American Society of Clinical Oncology
SYSUCC: Sun Yat-sen University Cancer Center
